# A Geographical Analysis of Emergency Medical Service Calls and Extreme Heat in King County, WA, USA (2007–2012)

**DOI:** 10.3390/ijerph14080937

**Published:** 2017-08-20

**Authors:** Aubrey C. DeVine, Phuong T. Vu, Michael G. Yost, Edmund Y. W. Seto, Tania M. Busch Isaksen

**Affiliations:** 1 Department of Environmental and Occupational Health Sciences, University of Washington, Seattle, WA 98105, USA; airion@uw.edu (M.G.Y.); eseto@uw.edu (E.Y.W.S.); tania@uw.edu (T.M.B.I.); 2 Department of Biostatistics, University of Washington, Seattle, WA 98105, USA; phuongvu@uw.edu

**Keywords:** extreme heat, climate change, emergency medical service calls, ambulance calls, community-level characteristics

## Abstract

This research analyzed the relationship between extreme heat and Emergency Medical Service (EMS) calls in King County, WA, USA between 2007 and 2012, including the effect of community-level characteristics. Extreme heat thresholds for the Basic Life Support (BLS) data and the Advanced Life Support (ALS) data were found using a piecewise generalized linear model with Akaike Information Criterion (AIC). The association between heat exposure and EMS call rates was investigated using a generalized estimating equations with Poisson mean model, while adjusting for community-level indicators of poverty, impervious surface, and elderly population (65+). In addition, we examined the effect modifications of these community-level factors. Extreme-heat thresholds of 31.1 °C and 33.5 °C humidex were determined for the BLS and ALS data, respectively. After adjusting for other variables in the model, increased BLS call volume was significantly associated with occurring on a heat day (relative rate (RR) = 1.080, *p* < 0.001), as well as in locations with higher percent poverty (RR = 1.066, *p* < 0.001). No significant effect modification was identified for the BLS data on a heat day. Controlling for other variables, higher ALS call volume was found to be significantly associated with a heat day (RR = 1.067, *p* < 0.001), as well as in locations with higher percent impervious surface (RR = 1.015, *p* = 0.039), higher percent of the population 65 years or older (RR = 1.057, *p* = 0.005), and higher percent poverty (RR = 1.041, *p* = 0.016). Furthermore, percent poverty and impervious surface were found to significantly modify the relative rate of ALS call volumes between a heat day and non-heat day. We conclude that EMS call volume increases significantly on a heat day compared to non-heat day for both call types. While this study shows that there is some effect modification between the community-level variables and call volume on a heat day, further research is necessary. Our findings also suggest that with adequate power, spatially refined analyses may not be necessary to accurately estimate the extreme-heat effect on health.

## 1. Introduction

According to the United States Climate and Health Assessment [1], the US annual average temperatures are predicted to rise at least 1.7 °C by the end of the century with chances of a 5.6 °C increase depending on many factors such as future emissions of greenhouse gases. With this temperature increase, will come more frequent and intense heat waves along with harmful effects to human health. Scientists predict for every doubling of carbon dioxide, it is likely that there will be a 1.9 to 4.5 °C increase in temperature [2,3]. Sherwood and Huber [4], argued a global mean increase of 7 °C would limit the habitability of certain regions in the world because areas exposed to 35 °C for extended periods of time will induce hyperthermia due the inability for dissipation of metabolic heat to occur. Extreme heat makes it difficult for the body to regulate its internal temperature increasing the potential for illnesses such as heatstroke and heat exhaustion along with negative consequences on chronic cardiovascular disease, respiratory disease and diabetes-related conditions [1]. As climate change increases global temperatures, an increase in heat-related morbidity and mortality is reasonably expected.

There are many studies that have analyzed the relationship between extreme heat and morbidity and mortality; nearly all have concluded that extreme heat is associated with increased risk of death, hospitalizations, and other illnesses, especially illnesses related to the cardiovascular and respiratory systems [5,6,7]. Furthermore, researchers have observed increased emergency medical service call volumes during heat events [8,9,10,11,12,13]. Bassil et al. [11] concluded, on average, there was a 16% increase in heat related illness calls for every one point increase in humidex. Dolney and Sheridan [12] calculated a 10% increase in ambulance calls on heat days compared to non-heat days. Additionally, Dolney and Sheridan [12] observed spatial variations in call volumes; the urban core experienced the greatest increase in call counts, while industrial and recreational areas had the greatest percent call volume increases on hot days.

Many environmental factors can influence heat burden in the general population. Environmental factors which influence local temperatures include buildings, open space, population density, normalized vegetation index (NDVI), vegetation, impervious surface, bare soil, and water proximity [14,15,16,17,18,19,20,21]. Vegetation and NDVI are strongly correlated with ground and air temperature [14,22,23,24,25,26]. As a result, increased vegetation is also associated with decreased heat stress and heat-related deaths [24,27,28]. More specifically, Elaisson and Svensson [29] used both environmental factors and weather patterns to find that on cloudy days, altitude and percent impervious surfaces/build-up were the two most important environmental factors explaining temperature variations, while on clear, calm days, distance from water explained most of the temperature variation, with sky-view factor, percent impervious surfaces, and percent vegetation as additional important factors.

Studies have also demonstrated that socioeconomic factors contribute to heat burden inequity. The highest extreme heat-related morbidity and mortality occur in cities and upon marginalized groups such as the poor, minority and elderly populations [30,31,32,33]. Socioeconomic factors associated with heat mortality include ethnic minorities, poverty, poor education, elderly (≥65 years), crime, and living alone [22,27,33,34,35,36]. Low income and minority groups are more likely to live in neighborhoods with high population densities, sparse vegetation, and little open space which significantly increases their exposure to heat [27]. To further exacerbate socioeconomic heat burden disparities, low income and minority communities generally do not have the same access to protective resources such as air conditioning, which has been found to be the most effective heat stress prevention factor [37,38,39]. Income level can affect the patient’s health status and ability to treat or manage pre-existing health conditions leaving them more susceptible to heat illness. For example, diabetes has a strong correlation with low income or poverty households [40] in addition to a strong correlation with extreme heat vulnerability [1,13]. Drug or alcohol use and the ability to seek help is another confounding factor correlated with both low socioeconomic status [22] and increased risk of an Emergency Medical Service (EMS) call in an extreme heat event [13]. It is important to consider these socioeconomic factors when comparing spatial distributions of extreme heat risk.

The Pacific Northwest is susceptible to the health effects of extreme heat. Research by Curriero et al. [41] determined that while cold temperatures have a greater effect on mortality in certain southern cities, heat exacerbated mortality in northern cities. Since southern cities generally experience more extreme heat than the colder climates of the north, neither region adapts well to opposite extreme temperatures. Reid et al. [42] specifically pointed out the Pacific Coast as one of the most vulnerable regions to extreme heat due to four factors: social and environmental vulnerability from education, race, and green space; social isolation; air conditioning prevalence; and the proportion of elderly and diabetes in the population. As the most populous northern county in the Pacific Northwest and 13th in the United States, research indicates that King County in Washington State is vulnerable to the health impacts of increasing extreme heat events. Specifically, Calkins et al. [13] found that in King County, WA, basic life support (BLS) calls increased by 8% on days exceeding a county-wide averaged maximum humidex above the 99th percentile (36.7 °C) and by 14% for advanced life support (ALS) calls on days exceeding a county-wide averaged maximum humidex above the 95th percentile (29.7 °C). Additionally, research from Busch Isaksen et al. [5,6] demonstrated increased risk for mortality and hospitalizations across age groups and cause of deaths and admissions. All of these previous studies, however, have used a county-wide averaged maximum daily humidex as their measurement of exposure.

This paper assesses the relative risk of higher rates of EMS calls on extreme heat days compared to non-heat days, accounting for social and environmental predictors for heat vulnerability including percent living in poverty, percent of the population over age 65, and percent impervious surface. This paper adds to the research of Calkins et al. [13] by redefining the spatial unit of exposure analysis from county-wide humidex averages to a 4 km by 7.5 km meteorological grid block scale to assess a local relative rate of EMS calls on extreme-heat days compared to non-heat days. Using local humidex measurements instead of county-wide averages further refined our extreme-heat threshold, thus reducing type II error from EMS call misclassification.

## 2. Materials and Methods

### 2.1. Exposure Data and Assessment

The University of Washington’s Climate Impacts Group provided meteorological data modeled on a 4 km by 7.5 km (1/16th degree resolution) block grid. These data were derived using the Parameter-elevation Relationships of Independent Slopes Model (PRISM) [43] and observations from the National Oceanic and Atmospheric Administration’s Global Historic Climate Network-Daily (GHCN) (Busch Isaksen et al. [5,6] explains the meteorological model further). Each grid block center point included daily minimum, maximum, and average temperature; relative humidity; and precipitation. From this, a minimum, maximum, and average humidex value for each day was calculated using the following equation:

(1) $$f\left(T,\;H\right)=T+\frac{5}{9}\times \left(v-10\right);\;v=\left(6.112+10^{\left[\frac{7.5T}{237.7}+T\right]}\right)\times \frac{H}{100}$$

where *T* is the temperature in Celsius, *H* is the humidity percentage, and *v* stands for the vapor pressure. Humidex is an apparent temperature index that describes the human perception of heat by taking the effects of humidity into account as well as air temperature. It represents a more accurate measure of heat burden than temperature alone [44].

### 2.2. EMS Data

The personnel of Seattle and King County Emergency Medical Services Division of the Department of Public Health prepared, de-identified, and supplied the University of Washington researchers with Emergency Medical Service call data. These data included information on EMS calls, both Basic Life Support (BLS) and Advanced Life Support (ALS) calls, from 2007 to 2012. During this time frame, thirty BLS agencies responded to an average of 165,000 BLS calls per year and six ALS agencies responded to an average of 45,000 ALS calls per year [45]. However, our analysis includes only the summer months, defined as May 1st to September 30th, of 2007–2012 which included 121,794 ALS calls and 441,119 BLS calls in King County, WA. The call classification was determined by the dispatcher based on the level of care they believed the patient needed from the information provided over the phone. However, it is important to note that despite whether an ALS unit responded, all calls in King County received a BLS response. BLS units send out emergency medical technician-trained firefighters who aid in non-invasive care, while ALS units are equipped with paramedics authorized for advanced patient care such as intubation, manual defibrillation, and intravenous medications to care for patients in critical condition who may need treatment before or during their transport to a medical facility. IRB approval for these data was granted from the University of Washington Human Subjects Division.

Each EMS call was assigned to a 4 by 7.5 km grid cell based on its call location. The few grid cells with missing center points were matched to the meteorological data from the nearest bordering center point. Data preparation excluded calls with a missing location variable, calls located on the outer border of the grid cell map, and cells with five or less total calls for the entire study period.

### 2.3. Demographic and Environmental Data

Population data by age [46] and poverty status [47] were downloaded from the US Census Bureau American Fact Finder database. These data were downloaded on the census tract level in Washington State for 2010, the midpoint of the study period. In order to extrapolate estimates to the grid cell scale, the intersect tool in ArcGIS found the percent of each census tract inside a given grid cell. These percentages were used as weights to find approximated population (total and by age groups) and percent poverty population for each grid cell.

Data for impervious surface were downloaded from the Washington State Department of Ecology [48] as raster data from remote sensing imagery techniques. These data were an estimate for land cover in 2006 and included a percentage value for each pixel in the county. From this, the average percent impervious surface was computed for each grid cell.

### 2.4. Statistical Methods

#### 2.4.1. Temporal Analysis on Extreme Heat Thresholds

We modified the approach in Busch Isaksen et al. [5] and Calkins et al. [13] to define the thresholds for our analysis. Calkins et al. [13] considered thresholds from among the 90th, 95th, and 99th percentiles of full year county-wide average humidex on the same meteorological dataset. Using a Poisson generalized linear model, Calkins et al. [13] picked a threshold that gave the best fit model in terms of Akaike Information Criterion (AIC) [49]. Due to various climates and landscapes throughout King County, heat index also varies, meaning that thresholds derived from county-wide average humidex could be misleading.

Instead, this study sought to analyze the relationship between heat exposure and EMS call volume on a finer spatial scale. We first used a Poisson generalized additive model (GAM) [50] to preliminarily evaluate the overall time trend as well as the relationship between log expected call volumes and humidex. Specifically, we modeled the effects of humidex and temporal trend of expected daily call count per each grid cell over the study period by penalized regression splines. We also accounted for population of each grid cell and adjusted for difference across days of the week.

To increase interpretability and ability to compare goodness of fit, we then simplified the non-parametric spline model with a crude piece-wise generalized linear model [51] with two knots. Based on overall trends observed from the GAM results, the first knot was set at the 25th percentile of all humidex values throughout the study period for the BLS dataset. For the ALS dataset, the first knot was set at the 50th percentile. The second knot, i.e. our extreme heat threshold of interest, was then identified by exploring 0.1 °C incremental changes starting at 25 °C and continuing through 40 °C. The chosen threshold $h^{*}$ is one that minimized the AIC of the following model:

(2) $$\log\mu_{ij}=\log p_{i}+\gamma_{0}+\gamma_{1}{\left(h_{ij}-h_{q}\right)}_{+}+\gamma_{2}{\left(h_{ij}-h^{*}\right)}_{+}+ns\left(t_{ij}\right)+\overset{7}{\underset{k=1}{\sum }}\gamma_{k}I_{\left\{day_{ij}=k\right\}}$$

where $\mu_{ij}$ is the expected daily call volume of grid cell *i* on day *j*, $\log p_{i}$ is the offset for population of cell *i*, $h_{q}$ is the 25th percentile of humidex for BLS data or 50th percentile of humidex for ALS data, $h^{*}$ is the optimal threshold to be chosen, and $\gamma_{k}$’s adjust for difference across days of the week. $ns\left(t_{ij}\right)$ is a natural cubic spline modeling the temporal trend of expected daily call count during the entire study period. Finally, ${\left(\ldots \right)}_{+}$ denotes the positive part of the expression evaluated in the parentheses. The GAM results were used to tune the degrees of freedom for the temporal spline terms.

#### 2.4.2. Association between Heat Exposure and EMS Call Rates

A day was then classified as a “heat day” if its local maximum humidex value exceeded the threshold derived from the temporal analysis. We investigated the association between heat exposure and daily EMS call volume by fitting a Poisson mean model:

(3) $$\log\mu_{ij}=\log p_{i}+\beta_{0}+\beta_{1}HD_{ij}+\beta_{2}Imp_{i}+\beta_{3}PopG65_{i}+\beta_{4}Pov_{i}+ns\left(t_{ij}\right)+\overset{7}{\underset{k=1}{\sum }}\beta_{k}I_{\left\{day_{ij}=k\right\}}$$

where $HD_{ij}$ is the indicator of a heat day, $Imp_{i}$ is the percentage of impervious surfaces of grid cell *i*, $PopG65_{i}$ is the percentage of population at 65 years old or older, and $Pov_{i}$ is the percentage of poverty. To account for temporal correlation across daily measurements on each grid cell, we employed the generalized estimating equations framework [52] where the model assumes an autoregressive working correlation structure among measurements within each grid cell. We adjusted for percentage of impervious surfaces, percentage of poverty, and percentage of population at 65 years old and older, because *a priori* we believed that these environmental and demographic factors could have possible implications on the expected daily call volumes. The exponentiated coefficient on the heat indicator represents the relative rate of daily call volumes between a heat day and a non-heat day.

We then constructed a time-average map for the expected RRs (relative rate) of daily BLS and ALS call volumes, separately, between a heat and non-heat day. To crudely detect any spatial correlation that the models were not designed to capture, we performed the Moran’s I Test [53] on the residuals calculated by subtracting the time-average observed RRs from the expected RRs estimated by the models.

#### 2.4.3. Effect Modification by Environmental and Demographic Characteristics

We proceeded to evaluate the possibility of effect modifications by these environmental and demographic variables. Effect modification was examined by adding interaction terms into the main model separately. An example of exploring effect modification is illustrated by the following model:

(4) $$\log\mu_{ij}=\log p_{i}+\alpha_{0}+\alpha_{1}HD_{ij}+\alpha_{2}Imp_{i}+\alpha_{3}PopG65_{i}+\alpha_{4}Pov_{i}+\alpha_{5}HD_{ij}\times Pov_{i}+ns\left(t_{ij}\right)+\overset{7}{\underset{k=1}{\sum }}\alpha_{k}I_{\left\{day_{ij}=k\right\}}$$

where the parameter of interest is $\alpha_{5}$, the coefficient on the interaction between heat exposure and the percentage of poverty. In this example, by testing the significance of this interaction term, we could identify whether the association between heat exposure and expected daily call volumes differ across areas with various level of poverty.

Statistical significance was set at level 0.05. Results for Section 2.4.2 and Section 2.4.3 were reported in terms of relative rates, i.e. exponentiated coefficients from Equations (3) and (4), to be scientifically meaningful. All statistical analyses were conducted using the R statistical analysis package version 3.4.0 [54].

## 3. Results

### 3.1. Statistical Results

Table 1 provides descriptive EMS data after removing grid cells with total count of five or less calls throughout the entire study period. After excluding cells with fewer than 5 calls total over the study period, we ended up with 124 grid cells to be included in the analysis for BLS data, and 116 grid cells to be included in the analysis for ALS data.

#### 3.1.1. Temporal Analysis of Extreme Heat Thresholds

**Figure 1:**
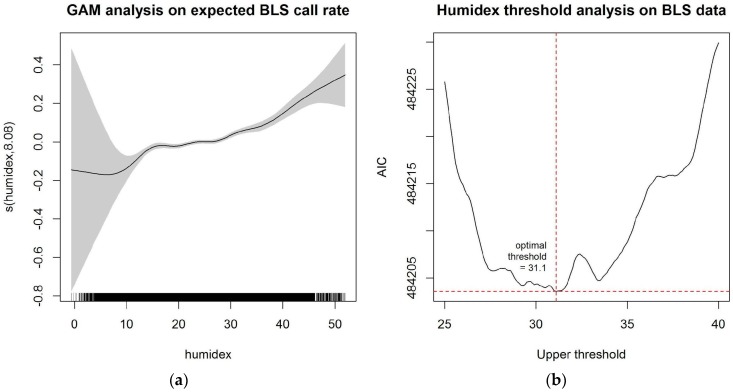
Temporal analysis on extreme heat threshold for BLS data: (**a**) Generalized additive model (GAM) with penalized regression spline of log expected daily BLS call rate and humidex; (**b**) Plot of AIC (Akaike Information Criterion) for the likelihood model when choosing optimal threshold between 25 °C and 40 °C.

The temporal analysis for BLS data, modeled by a penalized cubic regression spline, is reported in Figure 1. The relationship suggested a subtle increase in expected BLS call rate for humidex between 25 °C and 35 °C. When fitting a piecewise generalized linear approximation using two knots, the first knot was set at the 25th percentile of humidex, and a natural cubic spline with four degrees of freedom was used to model the time trend. The second knot was identified by exploring 0.1 °C incremental changes starting at 25 °C and continuing through 40 °C, and choosing one that minimized the AIC of the likelihood model. The chosen optimal threshold for BLS data was determined to be 31.1 °C.

**Figure 2:**
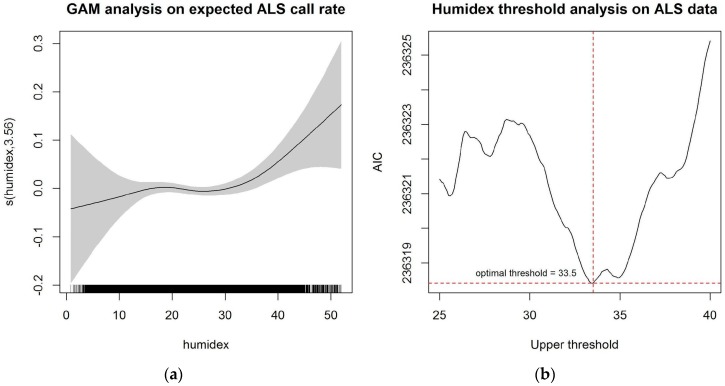
Temporal analysis on extreme heat threshold for ALS data: (**a**) Generalized additive model with penalized regression spline of log expected daily ALS call rate and humidex; (**b**) Plot of AIC for the likelihood model when choosing optimal threshold between 25 °C and 40 °C.

The temporal analysis for ALS data is reported in Figure 2. Based on the preliminary results, a natural cubic spline with five degrees of freedom was used in the piecewise linear approximation. The optimal threshold for ALS data was chosen to be 33.5 °C.

#### 3.1.2. Association between Heat Exposure and EMS Call Counts

Table 2 presents the statistical results for the analysis on the association between heat exposure and BLS call counts. Adjusting for cell population, various temporal effects, as well as percentages of impervious surfaces, population ≥ 65 years old, and percent living in poverty, the expected daily BLS call volume on a heat day, i.e. a day when local maximal humidex greater than 31.1 °C, was statistically significantly elevated, estimated to be 1.080 (95% CI: (1.060, 1.099)) times higher than the expected call volume on a non-heat day. The main effects of impervious surfaces and population ≥ 65 years old on the expected BLS call volume were not statistically significant at the 0.05 level. However, we estimated that a one percent increase in percent living in poverty was significantly associated with 1.066 (95% CI: (1.029, 1.105)) times the expected daily BLS call volume, controlling for other factors and time variables.

**Figure 3:**
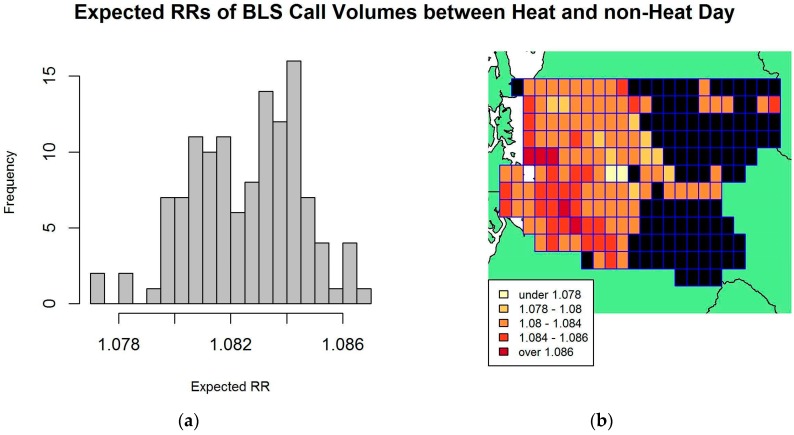
Relative rate (RR) analysis on BLS data: (**a**) Histogram of expected RRs of BLS call volumes between heat and non-heat day, defined by humidex threshold of 31.1 °C; (**b**) King County, Washington’s Grid map of expected RRs for BLS data, where black indicates grid cells without call data or too few call data to be included in the analysis. Each grid cell has size of 4 km by 7.5 km.

Using the fitted results from the model described in Table 2, we calculated and mapped the expected RRs of BLS call volumes between a heat day and a non-heat day across grid cells. The histogram and map of estimated RRs are displayed in Figure 3. The mean expected RR across 124 grid cells was 1.083, with a standard deviation of 0.002. Moran’s I test on the residuals did not find statistically significant evidence for spatial autocorrelation among neighboring grid cells.

Table 3 presents the statistical results for the analysis on the association between heat exposure and ALS call counts. Adjusting for cell population, various temporal effects as well as percentages of impervious surfaces, population ≥ 65 years old, and poverty, the expected daily ALS call volume on a heat day, i.e. a day when local maximal humidex greater than 33.5 °C, was estimated to be 1.067 (95% CI of (1.035, 1.100)) times higher than the expected ALS call volume on a non-heat day. This result was statistically significant with a *p*-value of less than 0.001. Controlling for other factors and time variables, we estimated that a one percent increase in impervious surfaces was associated with 1.015 (95% CI of (1.001, 1.029)) times the expected daily ALS call volume. Similarly, a one percent increase in population ≥ 65 years old was associated with 1.057 (95% CI of (1.017, 1.098)) times increase in the expected daily ALS call volume, and a one percent increase in poverty translated to 1.041 (95% CI of (1.008, 1.076)) times increase in the expected daily ALS call volume.

**Figure 4:**
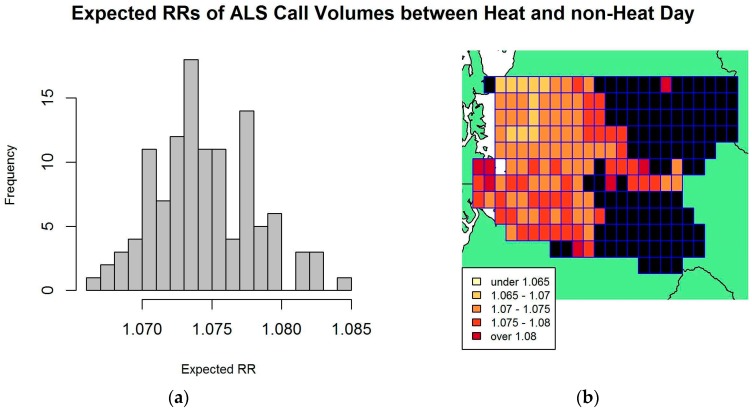
Relative rate (RR) analysis on ALS data: (**a**) Histogram of expected RRs of ALS call volumes between heat and non-heat day, defined by humidex threshold of 33.5 °C; (**b**) King County, Washington’s Grid map of expected RRs for ALS data, where black indicates grid cells without call data or too few call data to be included in the analysis. Each grid cell has size of 4 km by 7.5 km.

Similarly, we used the fitted results from the model described in Table 3 to calculate and map the expected RRs of ALS call volumes between a heat day and a non-heat day across grid cells. The histogram and map of estimated RRs are displayed in Figure 4. The mean expected RR across 124 grid cells was 1.074, with a standard deviation of 0.004. Again Moran’s I test on the residuals did not find statistically significant evidence for spatial autocorrelation among neighboring grid cells.

#### 3.1.3. Effect Modifications by Environmental and Demographic Characteristics

We found no evidence that the relative rate of BLS call volumes between a heat day and non-heat day differed by percent impervious surfaces, percent population ≥ 65 years old, or percent poverty. The results are reported in Table A1 of the Appendix. The statistical results for three separate models investigating the effect modifications by the community-level characteristics for the ALS data are presented in Table 4. We found no evidence that the relationship between heat exposure and ALS call volume was modified by the percentage of population ≥ 65 years old.

The exact interpretation of the interaction term is tricky given the continuous nature of the environmental and socioeconomic variables, and thus we attempted to give numerical examples as followed. In the model that tested the effect modification of percent poverty, for example, among grid cells with 5% living in poverty (41st percentile of percent poverty for all available grid cells), the relative rate of ALS calls between a heat day and non-heat day was estimated to be 1.150, i.e. daily ALS volume on a heat day is 1.150 times higher than the volume on a non-heat day. This value was derived from Equation (4) by exponentiating $\left(\alpha_{1}+5\alpha_{5}\right)$. However, among grid cells with 10% living in poverty (around 84th percentile), this relative rate was estimated to be only 1.080 using the same calculation method. Similarly, in the model that tested the effect modification of percent impervious surfaces, among grid cells with 15% percent impervious surfaces, the relative rate of ALS calls between a heat day and non-heat day was estimated to be 1.133. Meanwhile, among grid cells with 20% percent impervious surface, the relative rate was estimated to be only 1.115. The results essentially suggested that the relative rate of daily ALS call volumes between a heat day and a non-heat day differ across various level of impervious surfaces or percent poverty.

## 4. Discussion

Using a finer spatial scale to assess heat exposure, this analysis identified an extreme heat-day threshold of 31.1 °C for BLS and 33.5 °C for ALS calls. Compared to the county-wide derived thresholds from Calkins et al. [13], this study’s extreme-heat day threshold increased for BLS calls from 29.7 °C to 31.1 °C humidex, but decreased for ALS calls from 36.7 °C to 33.5 °C humidex. Despite the adjustments, the threshold of ALS calls remains higher than that of BLS calls, continuing to support the hypothesis that higher temperatures are associated with more severe patient conditions requiring more involved interventions.

**Figure A1:**
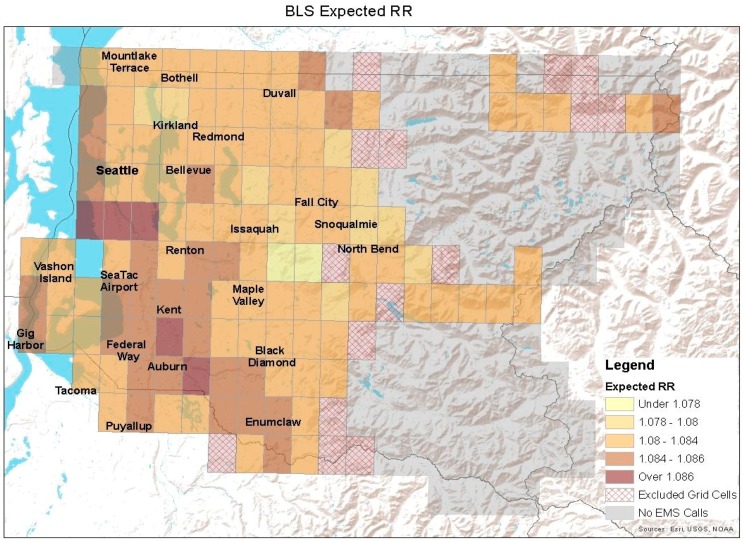
King County, Washington’s Grid map of expected RRs for BLS data with local city labels, where red checkers indicate grid cells without call data or too few call data to be included in the analysis. Each grid cell has size of 4 km by 7.5 km.

**Figure A2:**
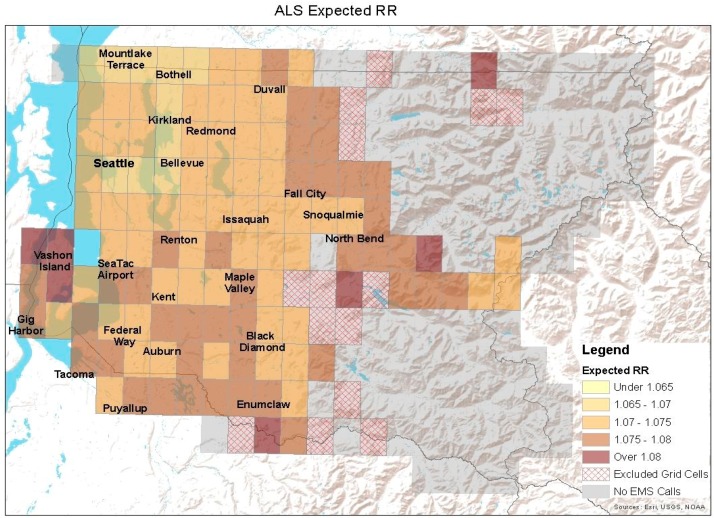
King County, Washington’s Grid map of expected RRs for ALS data with local city labels, where red checkers indicate grid cells without call data or too few call data to be included in the analysis. Each grid cell has size of 4 km by 7.5 km.

In the Calkins et al. [13] study using the county-wide average humidex to assess exposure, a RR of 1.08 (95% CI 1.06, 1.09) was observed for BLS calls and 1.14 (95% CI 1.09, 1.2) for ALS calls, on a heat day compared to a non-heat day. After refining our exposure assessment, and adjusting for community-level characteristics, our analysis observed the same 1.08 (95% CI: 1.060, 1.0) RR for BLS calls, but a much lower 1.067 RR for ALS calls on heat days compared to non-heat days. The difference between study results for ALS call risk could be attributed to effects from community-level characteristics and improved study power. However, it is more likely that the higher extreme heat threshold in Calkins et al. [13], 36.7 °C humidex resulted in categorizing and analyzing fewer extreme-heat days compared to this study’s extreme heat threshold of 33.5 °C humidex. There were 23 heat days in Calkins et al. [13], and 28 99th percentile heat days in this analysis. The lower number of extreme heat days may have reduced study power and resulted in a higher overall risk for ALS calls in Calkins et al. [13]. On the other hand, study power and the overall results for the BLS dataset remain strong in the county-wide analysis which included 112 heat days [13] and this spatially refined analysis which included 149 95th percentile heat days. This suggests that when there is enough study power, the refined analysis may not be necessary to accurately estimate the risk of EMS calls in the region. The geographical distribution of expected RRs for BLS calls on an extreme heat day compared to a non-heat day can be found in Figure A1 of the Appendix and the distribution of expected RRs for ALS calls in Figure A2 of the Appendix.

Furthermore, this study found a significant positive association between BLS call volumes and only one of the community-level characteristics: percent poverty. This suggests that, in general, more BLS calls are expected in areas with higher percent poverty. However, the interaction term between heat day and poverty was not statistically significant, meaning there was no statistical evidence that BLS call volumes increased with percent poverty on extreme heat days compared to non-heat days. This finding is contrary to previous studies which indicate individuals living in poverty have fewer opportunities to access air conditioning, clean drinking water, and health care, which may increase their vulnerability to heat [37,38,39]. With less access to healthcare, those in poverty are more susceptible to underlying health conditions which can make them more vulnerable to health complications during extreme heat events. For example, diabetes, has been shown to strongly correlate with households below the poverty-line [40] in addition to extreme heat vulnerability [1,13]. Further research should continue to examine the potential relationships between poverty, heat and BLS calls, including whether there may be coping mechanisms specific to poor populations in this region that might account for the lack of statistically significant interaction.

This study also found a significant positive association between expected ALS call volumes and all of the community-level characteristics analyzed: percent impervious surface, percentage living in poverty, and percent of the population age 65 or older. Daily ALS call volumes increased in areas with increased poverty, higher populations of elderly residents, and higher percentages of impervious surfaces. Moreover, poverty and impervious surface have a significant negative interaction term with heat day. This indicates that as percent poverty or percent impervious surface increases, ALS call volume decreases on an extreme heat day compared to a non-heat day. These negative relationships are also contradictory of previous literature. Just as BLS call volumes are expected to increase, research also indicates that ALS call volume would increase as poverty increases on an extreme heat day compared to a non-heat day. In addition, past research also suggests that ALS call volume should increase as impervious surface increases. Akbari [55] found an increase in percent impervious surface and percent development in urban cores can lead to an increase by an additional 5.5 °C (22 °F) in cities compared to rural areas nearby, which would increase one’s susceptibility to heat illness. Conversely, an increase in tree canopy was shown to cool surrounding air temperatures during an extreme heat event [14,22,23,24,25,26] which would decrease one’s susceptibility to heat illness. The model results do suggest some effect modification exists, but the effect modification is non-intuitive, and it is possible that an additive model is not enough to fully understand the effect modification. Further research is needed to explore relationships with the community-level variables, as well as the preventative influence that active public health outreach and preparedness programs are having on our vulnerable communities’ health outcomes.

There are several limitations to this research. Grid cell population data were available from only one of the six years in the study time frame, so we could not account for natural demographic, environmental, or socioeconomic changes over time. The extrapolation of population and poverty from the census tract level to the grid cell level, assumed population and poverty were evenly distributed throughout census tracts. This extrapolation from the census tracts to the grid cells contributes greater uncertainty in the more rural eastern side of the county where census tracts are large and encompassed multiple grid cells with sparse populations.

Although we adjusted for temporal trends by including temporal splines and indicators for day of the week, there may be others factors that were not accounted for due to a lack of data. For example, information pertaining to time spent within grids and moving between grids (residential versus employment movement patterns) could be potential time trend variables affecting call counts over the six-year period. Additionally, there could be monthly trends which were not included, or undetectable spatial correlations that were not picked up on by a crude Moran’s I test.

This study included no information regarding individual patient’s underlying health history, activities before/during the EMS call, environment the patient was located in, or the patient’s specific socioeconomic status. These regional data on poverty, elderly population and impervious surface were not case specific, but rather were the averages of the population over the grid cell for the call location. Therefore, it is possible the patient did not live in the same grid cell they called for an ambulance, in which case, these grid cell data of their location may not adequately reflect the average of their community. Future research should collect patient-specific information.

Despite these limitations, our analysis has several potential practical implications for managing EMS calls during extreme temperature days in King County. Already, we have communicated preliminary results to those who have provided us with the EMS data, in order to explore strategies to build awareness of the potential for increased call volume, and prioritization services to areas with communities that are most vulnerable to heat effects. Particularly for more severe ALS calls, reducing response times may have a dramatic impact on the outcomes of these calls. Longer term strategies, such as building awareness and adaptive capacity to protect against extreme heat in poor, older, and urban populations will be an important aspect of developing climate action plans for this region. We would expect with climate change that the prevalence of extreme heat days may increase in this region, making such emergency service planning more urgent. Furthermore, a comparison of the county wide analysis by Calkins et al. [13] and this analysis suggests that in future research with adequate study power, a refined spatial analysis within counties may not be necessary to accurately portray the effects of extreme heat on EMS call volumes.

## 5. Conclusions

Building off previous research, this paper has a more accurate exposure assessment that potentially addresses exposure misclassification that existed in the previous paper. In addition, we re-analyzed the association between extreme heat and EMS calls adjusting for percent poverty, percent impervious surface, and percent of the population 65 years or older. On an extreme heat day, we continued to find increased BLS and ALS call volumes compared to non-heat days. While the BLS risk was the same between this spatially refined analysis and a county wide analysis, the ALS risk was much lower in the refined analysis. Future research should explore a larger EMS dataset as well as more patient specific variables.

## Figures and Tables

**Table 1:** Descriptive statistics for EMS (Emergency Medical Service) call volumes.

Variable	BLS Call	ALS Call
Total number of calls—raw data	441,119	121,794
Total number of calls included in statistical analysis	434,853	120,638
Number of grid cells included in statistical analysis	124	116
Average number of local heat days per grid cell ^1^	109.08	60.44
Average number of local non-heat days per grid cell ^1^	808.90	857.60
Average (observed) number of calls per heat day per grid cell (SD)	4.16 (7.51)	1.24 (2.16)
Average (observed) number of calls per non-heat day per grid cell (SD)	3.78 (6.77)	1.13 (1.99)

Note: ^1^ A day was classified as a heat day if the local maximal humidex value exceeded a pre-specified threshold. The extreme heat threshold was chosen to be 31.1 °C for BLS (Basic Life Support) calls and 33.5 °C for ALS (Advanced Life Support) calls.

**Table 2:** Relative rate results: association between heat exposure (heat day versus non-heat day) and daily BLS call volume, adjusting for community-level factors ^1^.

Variable	Estimated RR	95% Confidence Interval	*p* Value
Heat day	1.080	(1.060, 1.099)	<0.001
% Impervious surfaces	1.011	(0.998, 1.025)	0.102
% Population ≥ 65 years old	1.033	(0.984, 1.085)	0.185
% Poverty	1.066	(1.029, 1.105)	<0.001

Note: ^1^ The model adjusted for temporal trend in expected BLS call counts by including indicators for days of the week and a natural cubic spline for time with four degrees of freedom. RR: relative rate.

**Table 3:** Relative rate results: association between heat exposure (heat day versus non-heat day) and daily ALS call volume, adjusting for community-level factors ^1^.

Variable	Estimated RR	95% Confidence Interval	*p* Value
Heat day	1.067	(1.035, 1.100)	<0.001
% Impervious surfaces	1.015	(1.001, 1.029)	0.039
% Population ≥ 65 years old	1.057	(1.017, 1.098)	0.005
% Poverty	1.041	(1.008, 1.076)	0.016

Note: ^1^ The model adjusted for temporal trend in expected ALS call counts by including indicators for days of the week and a natural cubic spline for time with five degrees of freedom.

**Table 4:** Relative rate results: effect modifications of the environmental and demographic factors on the association between heat exposure and daily ALS call counts ^1^.

Variable	Estimated RR	95% Confidence Interval	*p* Value
Heat day	1.190	(1.093, 1.295)	<0.001
% Impervious surfaces	1.015	(1.001, 1.029)	0.037
% Population ≥ 65 years old	1.057	(1.017, 1.098)	0.005
% Poverty	1.041	(1.008, 1.076)	0.016
Interaction between heat and % impervious surfaces	0.997	(0.994, 0.999)	0.007
Heat day	1.006	(0.855, 1.184)	0.941
% Impervious surfaces	1.015	(1.001, 1.029)	0.039
% Population ≥ 65 years old	1.056	(1.016, 1.098)	0.005
% Poverty	1.041	(1.008, 1.076)	0.016
Interaction between heat and % population ≥ 65 years old	1.005	(0.992, 1.019)	0.446
Heat day	1.225	(1.130, 1.327)	<0.001
% Impervious surfaces	1.015	(1.001, 1.029)	0.038
% Population ≥ 65 years old	1.057	(1.017, 1.098)	0.005
% Poverty	1.042	(1.008, 1.077)	0.014
Interaction between heat and % poverty	0.988	(0.981, 0.994)	<0.001

Note: ^1^ The model adjusted for temporal trend in expected ALS call counts by including indicators for days of the week and a natural cubic spline for time with five degrees of freedom.

## Appendix

**Table A1:** Relative rate results: effect modifications of the environmental and demographic factors on the association between heat exposure and daily BLS call counts ^1^.

Variable	Estimated RR	95% Confidence Interval	*p* Value
Heat day	1.102	(1.054, 1.151)	<0.001
% Impervious surfaces	1.011	(0.998, 1.025)	0.101
% Population ≥ 65 years old	1.033	(0.984, 1.085)	0.184
% Poverty	1.066	(1.029, 1.105)	<0.001
Interaction between heat and % impervious surfaces	0.999	(0.998, 1.001)	0.353
Heat day	1.097	(0.992, 1.215)	0.072
% Impervious surfaces	1.011	(0.998, 1.025)	0.102
% Population ≥ 65 years old	1.034	(0.984, 1.085)	0.187
% Poverty	1.064	(1.029, 1.105)	<0.001
Interaction between heat and % population ≥ 65 years old	0.999	(0.989, 1.008)	0.752
Heat day	1.123	(1.066, 1.182)	<0.001
% Impervious surfaces	1.011	(0.998, 1.025)	0.102
% Population ≥ 65 years old	1.033	(0.985, 1.085)	0.184
% Poverty	1.067	(1.029, 1.106)	<0.001
Interaction between heat and % poverty	0.997	(0.992, 1.001)	0.144

Note: ^1^ The model adjusted for temporal trend in expected BLS call counts by including indicators for days of the week and a natural cubic spline for time with four degrees of freedom.
